# Phylogenetic Analysis and Molecular Evolution Patterns in the *MIR482-MIR1448* Polycistron of *Populus* L

**DOI:** 10.1371/journal.pone.0047811

**Published:** 2012-10-18

**Authors:** Jia-Ping Zhao, Shu Diao, Bing-Yu Zhang, Bao-Qing Niu, Qing-Ling Wang, Xian-Chong Wan, You-Qing Luo

**Affiliations:** 1 State Key Laboratory of Tree Genetics and Breeding, Institute of New Forestry Technology, Chinese Academy of Forestry, Beijing, China; 2 Key Laboratory for Silviculture and Conservation, Ministry of Education, Beijing Forestry University, Beijing, China; 3 State Key Laboratory of Tree Genetics and Breeding, Research Institute of Forestry, Chinese Academy of Forestry, Beijing, China; 4 Agricultural University of Hebei, Baoding, China; International Centre for Genetic Engineering and Biotechnology, Italy

## Abstract

The microRNAs (miRNAs) miR482 and miR1448 are disease resistance-related miRNAs; the former is ubiquitously distributed in seed plants whereas the latter has only been reported in *Populus trichocarpa*. The precursor and mature sequences of poplar miR1448 are highly homologous to those of poplar miR482, and these two miRNAs are located in one transcript as a polycistron. Therefore, we hypothesized that the *MIR1448* gene may have evolved from the *MIR482* gene in poplar. However, the molecular evolution patterns of this process remain unclear. In this study, utilizing cloning and Blast analysis in NCBI ESTs and whole-genome shotgun contigs (WGS) dataset, we determined that the *MIR482-MIR1448* polycistron is a family-specific clustered miRNA in Salicaceae. Moreover, phylogenetic analysis illustrated that *MIR1448* is the product of a tandem duplication event from *MIR482*. Nucleotide substitution analysis revealed that both *MIR482* and *MIR1448* have more rapid evolution ratios than ribosomal DNA (*rDNA*) genes, and that compensatory mutations that occurred in the stem region of the secondary structure were the main mechanisms that drove the evolution of these *MIRNA* genes. Furthermore, by comparing the substitution patterns in the miRNA-target complexes of miR482 and miR1448, we inferred that co-evolution between miRNAs and their targets was the major force that drove the “duplicated *MIR482*” evolve to *MIR1448*. We propose a novel miRNA-target pairing pattern called the “frameshift targeted mechanism” to explain the gain of target genes by miR1448. The results also imply that the major role of miR482 was in resistance to disease or other stresses via NBS-LRR proteins, whereas the biological functions of miR1448 are more diverse.

## Introduction

MicroRNAs (miRNAs) are a family of short (about 20∼22 nucleotides in length) non-coding RNA molecules that mediate repressive gene regulation through RNA silencing at the post-transcriptional level in plants and animals [1]. In addition to their contribution to plant growth, development, and metabolism, miRNAs are integral components of plant responses to adverse abiotic stresses [1], [2], [3].

Recent researches have illustrated that miRNAs are involved in the response of plants to bacterial and fungal pathogens. For example, Chiang and his colleagues cloned and identified 26 miRNAs that responded to the fusiform rust pathogen (*Cronartium quercuum* f. sp. *fusiforme*) in the stem xylem of loblolly pine (*Pinus taeda*) [4]. There were 24 miRNAs responsive to the pathogen of powdery mildew (*Erysiphe graminis* f. sp. *Tritici*) in wheat leaves [5]. We previously found that 12 miRNA families responded to the poplar canker pathogen (*Botryosphaeria dothidea* Ces. & De Not.) in *Populus trichocarpa* (unpublished data). Among these, one species-specific miRNA, miR1448, was of interest regarding the biological functions of its target genes. Bioinformatics analysis and experimental detection validated that poplar miR1448 could target some nucleotide-binding site leucine-rich repeat (NBS-LRR) protein-encoding genes, suggesting that it might play an important role in plant resistance to biotic and abiotic stresses [6]. Because miR1448 is species-specific and has not been reported in any other plant species, it has been assumed to be a “young miRNA” [7] that has only recently become involved in the interaction between poplars and their environment during the long evolutionary history of this species. Therefore, the mechanism of its origin and its molecular evolution pattern is an interesting issue in plant pathological research.

**Figure 1:**
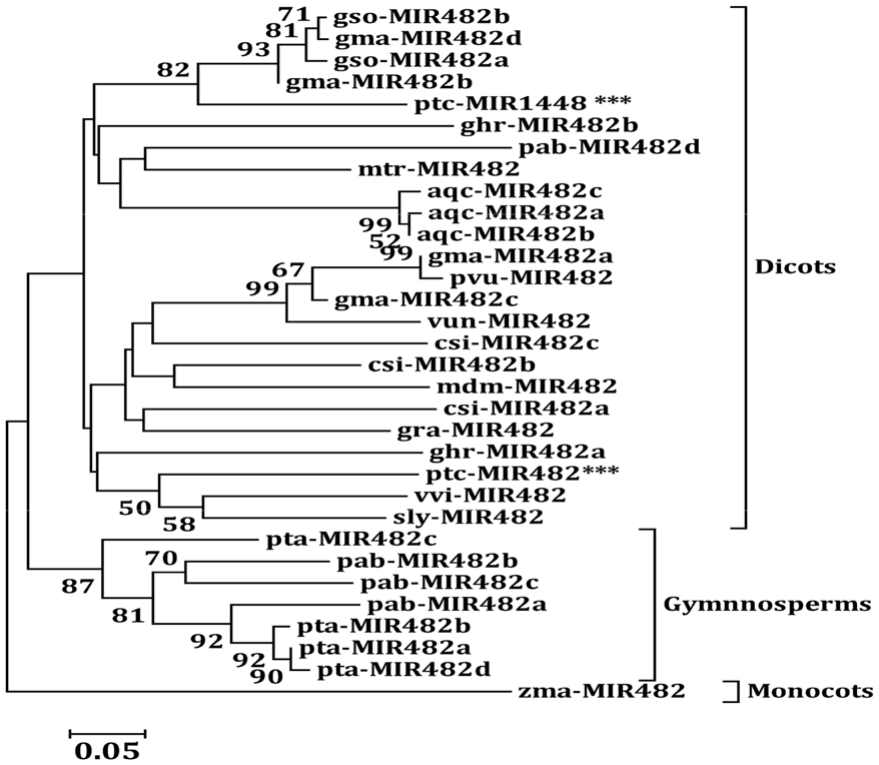
Neighbor-joining phylogenetic tree of pre-miR482 sequences in seed plants obtained from MEGA 5.0. Numbers in the tree represent the bootstrap value (bootstrap values below 50% are not shown at the nodes). The phylogeny of pre-miR1448 of *P. trichocarpa* is also compared to pre-miR482 sequences in other plants.

Interestingly, we recently identified another disease resistance-related miRNA, miR482, clustered with miR1448 in a narrow region (272 nt) of the poplar chromosome LG_VIII in the preliminary study of this research. MiR482 was ubiquitously distributed in many plants and it also targeted some NBS-LRR protein-encoding genes by target their P-loop motifs [8]. We also found that *miR1448* and *miR482* genes were both located in 12 expression sequence tags (ESTs) of approximately 600 base pairs (bp) in length from four poplar species or hybrids. This confirms that there is a *MIR482-MIR1448* polycistron [9], [10] in poplars. Finally, alignment analysis revealed that both the precursor and mature sequences of these two miRNAs are highly homologous.

**Table 1:** Plant materials used in this study.

Number	Species	Section of *Populus*	Families	Location*	GenBank Accession Number	
					rDNA-ITS	miR482-miR1448
P01	*Populus alba* L.	Sect. *Leuce* Duby	*Salicaceae*	CAF	JQ898621	JQ898652
P02	*P. deltoides* Bartr. 174-1-1	Sect. *Aigeiros* Dub	*Salicaceae*	CAF	JQ898622	JQ898653
P03	*P. yunnanensis* Dode	Sect. *Tacamahaca* Spach	*Salicaceae*	CAF	JQ898623	JQ898654
P04	*P. deltoides* Bartr.	Sect. *Aigeiros* Duby	*Salicaceae*	CAF	JQ898624	JQ898655
P05	*P. szechuanica* Schneid	Sect. *Tacamahaca* Spach	*Salicaceae*	CAF	JQ898625	JQ898656
P06	*P. cathayanna* Rehd.	Sect. *Tacamahaca* Spach	*Salicaceae*	CAF	JQ898626	JQ898657
P07	*P. simonii* Carr.	Sect. *Tacamahaca* Spach	*Salicaceae*	BBG	JQ898627	JQ898658
P08	*P. lasiocarpa* Oliv.	Sect. *Leucoides* Spach	*Salicaceae*	CAF	JQ898628	JQ898659
P09	*P. ussuriensis* Kom.	Sect. *Tacamahaca* Spach	*Salicaceae*	CAF	JQ898629	JQ898660
P10	*P. deltoides* Bartr. LA09-N1	Sect. *Aigeiros* Duby	*Salicaceae*	CAF	JQ898630	JQ898661
P11	*P. nigra* L. Wuhe 008	Sect. *Aigeiros* Duby	*Salicaceae*	CAF	JQ898631	JQ898662
P12	*P. deltoides* Bartr. Shanghaiguan23	Sect. *Aigeiros* Duby	*Salicaceae*	CAF	JQ898632	JQ898651
P13	*P. nigra* L. N77	Sect. *Aigeiros* Duby	*Salicaceae*	CAF	JQ898633	JQ898663
P14	*P. maximowiczii* A. Henry kuandian Q11	Sect. *Tacamahaca* Spach	*Salicaceae*	CAF	JQ898634	JQ898664
P15	*P. euphratica* Oliv.	Sect. *Turanga* Bge.	*Salicaceae*	CAF	JQ898635	JQ898665
P16	*P. trichocarpa* Torr.	Sect. *Tacamahaca* Spach	*Salicaceae*	CAF	JQ898636	JQ898666
P17	*P. devidiana* Dode.	Sect. *Leuce* Duby	*Salicaceae*	CAF	JQ898637	JQ898667
P18	*Idesia polycarpa* Maxim	-	*Salicaceae*	BBG	JQ898638	JQ898668
P19	*Idesia polycarpa* Maxim	-	*Salicaceae*	BBG	JQ898639	JQ898669
P20	*Carallia diplopetala* Hand.-Mazz.	-	*Rhizophoraceae*	BBG	-	-
P21	*Salix matsudana* cv. Pendula Koidz.	-	*Salicaceae*	CAF	JQ898640	JQ898670
P23	*P. nigra* L.	Sect. *Aigeiros* Duby	*Salicaceae*	CAF	JQ898641	JQ898671
P24	*P. hopeiensis* Hu et Chow	Sect. *Leuce* Duby	*Salicaceae*	CAF	JQ898642	JQ898672
P26	*Salix matsudana* Koidz.	-	*Salicaceae*	CAF	JQ898643	JQ898673
P27	*P. tomentosa* Carr.	Sect. *Leuce* Duby	*Salicaceae*	CAF	JQ898644	JQ898674
P28	*P. deltoides* Bartr.	Sect. *Aigeiros* Duby	*Salicaceae*	BBG	JQ898645	JQ898675
P29	*Vernicia fordii* (Hemsl.) Airy Shaw	-	*Euphorbiaceae*	BBG	JQ898646	-
P30	*Euphorbia neriifolia* L.	-	*Euphorbiaceae*	BBG	JQ898647	-
P31	*Codiaeum variegatum* (L.) A. Juss.	-	*Euphorbiaceae*	BBG	JQ898648	-
P32	*P. cathayanna* Rehd.	Sect. *Tacamahaca* Spach	*Salicaceae*	CAF	JQ898649	JQ898676

* Chinese academy of forestry (CAF), Beijing Botanical Garden (BBG).

Therefore, we hypothesized that poplar *MIR1448* might have evolved from *MIR482* through tandem duplication events. However, the mechanisms in *Populus* that drove the “duplicated miR482” evolve to *miR1448* is still not clear. To verify this hypothesis, and to reveal the potential evolutionary mechanism of *MIR1448*, we considered a phylogenetic analysis of plant *MIR482*, the thermodynamic stability of its secondary structure, nucleotide substitution models, a compensatory substitution model for the stem region, and the possible expression patterns of the *MIR482* and *MIR1448* genes.

**Figure 2:**
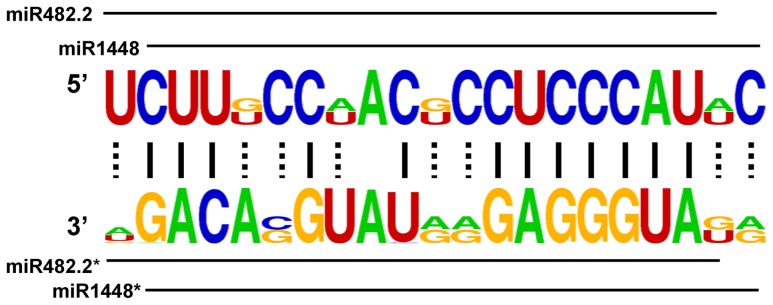
The nucleotide sequences of miR482 and miR1448 in *Populus* in the miRNA:miRNA* duplex.

## Results

### *MIR482* is a highly diverse miRNA gene that is ubiquitously distributed in gymnosperm, monocot, and dicot plants

The mature sequences and their corresponding precursor sequences of the total 31 *MIR482* gene that distributed in 16 gymnosperm, dicot, and monocot plants were derived from miRBase release 18. Of these, 7 were identified as single-copy genes in cotton, cowpea, maize, *Medicago truncatula*, poplar, tomato, and grape, whereas the other 24 were found in 9 plant species as part of the miRNA family. Substantial diversity was found in the nucleotide sequences of pre-miR482 within the plants. For example, the length of pre-miR482 varied from 69 to 179 nt (pre-miR482b of soybean and pre-miR482a of *Aquilegia*, respectively) and the average distance (p-distance) was as high as 0.401.

The results of the phylogenetic analysis (Figure 1) indicate that *MIR482* is an “old miRNA” gene [7], and its formation in plants predates the split of gymnosperms and angiosperms (∼300 million years ago) [11]. In addition, the paralog genes of *MIR482* in some specific species (such as *MIR482a*, −*b*, −*c* and −*d* in loblolly pine) are the products of one or two gene duplication events after the species formed.

### The *MIR482-MIR1448* polycistron is specific to plants in the family Salicaceae

We attempted to amplify the *MIR482-MIR1448* polycistron in 30 plants belonging to the families Salicaceae, Euphorbiaceae, and Rhizophoraceae using five forward primers and seven reverse primers. However, only 26 plants (including 2 *Idesia*, 22 *Populus*, and 2 *Salix*) in Salicaceae could be amplified (Table 1). Moreover, with the exception of poplars, none of the *MIR482-MIR1448* homologous sequences or miR482-related polycistron was derived from the NCBI EST database and genome database of other plant species. These results imply that the clustered *MIR482-MIR1448* genes are family-specific miRNA genes found only in Salicaceae.

By comparing the pre-miR482, pre-miR1448, and the mature sequences of miR482 and miR1448 in *P. trichocarpa*, we identified the precursors and mature sequences of miR482 and miR1448 in Salicaceae (poplar pre-miR482 produced two mature miRNAs, miR482.1 and miR482.2, which shared 15 overlapping nucleotides). The more homologous relationship between miR1448 and miR482.2 is discussed below.

The main characteristics of the Salicaceae *MIR482* and *MIR1448* genes are as follows. The mature miR482.2 and mature miR1448 in the genera *Salix*, *Populus*, and *Idesia* were identical, being 5′UCUUGCCUACUCCUCCCAUU3′ and 5′CUUUCCAACGCCUCCCAUAC 3′, respectively. The pre-miR482 sequences were 107 nt and 109 nt in length in *Populus* and *Salix*, respectively. The pre-miR1448 sequences were 85 nt in *Populus* and *Salix*. Both pre-miR482 and pre-miR1448 were highly conserved in *Populus* and *Salix*, and the nucleotide divergence was 0.0206 and 0.0269 (p-distance), respectively. As with *P. trichorarpa*, one 80 nt internal region was also located between these two precursors. Finally, compared to the other regions in the sequences of the *MIR482-MIR1448* polycistron, the upstream sequences before −92 nt of pre-miR482 are highly diverse. Similar to the other protein-coding genes, the upstream sequences of the *MIR482-MIR1448* polycistron consisted of some AT-rich regions. In addition, some deletions of long DNA fragments (∼240 nt) always appeared in the upstream sequences of the *MIR482-MIR1448* polycistron from the various different species, and even from within the same species.

### *MIR1448* is the tandem duplicate product of *MIR482* in Salicaceae

The phylogenetic analysis (Figure 1) also suggested that pre-miR1448 sequences in *P. trichocarpa* were homologous to pre-miR482 sequences not only in *P. trichocarpa* but also in other plants. LOGO representation indicated that nucleotide sequences in the miRNA:miRNA* duplex between miR482.2 and miR1448 were highly conserved. For example, 15 nucleotide sites were identical in miR482.2 and miR1448, while 13 were identical in miR482.2* and miR1448* (Figure 2). Additionally, PCR amplification and Blast analysis demonstrated the *MIR482-MIR1448* polycistron structure is only existed in Salicaceae plants.

From the results presented above, we inferred that the *MIR1448* gene was produced by tandem replication of the *MIR482* gene, and that this replication event might occurred predated the split of the Salicaceae family (∼60 to 65 million years ago) [12].

### Secondary structure and thermodynamic profiling of the *MIR482* and *MIR1448* genes

**Figure 3:**
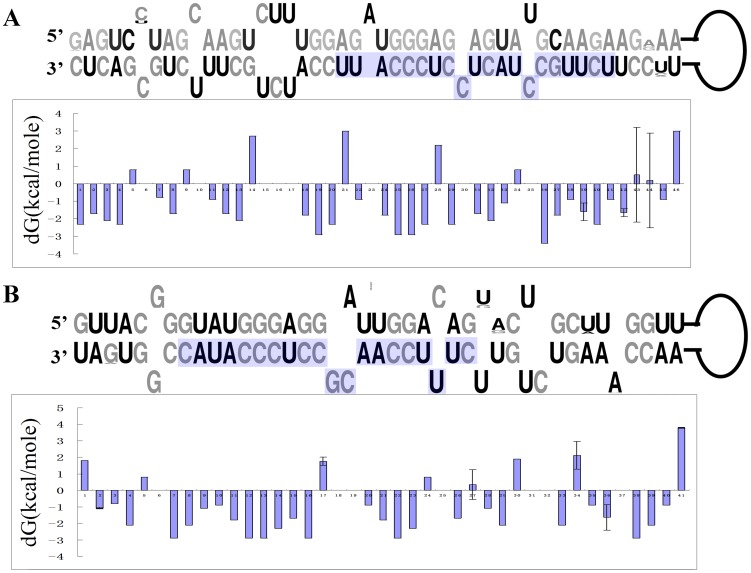
The stem-loop structure and thermodynamic stability of the pre-miR482 and pre-miR1448 in *Populus*. Free energy values are given in kcal/mole. Vertical bars indicate between-species variability calculated as the double standard error. The mature sequences of miR482.2 and miR1448 were highlighted in light blue.

The precursor and mature sequences of miR482.2 and miR1448 were determined for the highly homologous *MIR482-MIR1448* polycistron in *Populus* and *Salix*, according to the aligned results. The pre-miR482 and the pre-miR1448 in both *Populus* and *Salix* could form the classic stem-loop structures that closely resemble the secondary structures of pre-miR482 and pre-miR1448 in *P. trichocarpa* respectively [6], [13] (Figure 3). The minimum free energy (MFE) analysis indicated that the stability of the secondary structure of pre-miR1448 in both *Populus* and *Salix* was −42.6±1.75 kcal/mole and that of pre-miR482 was −45.3±2.12 kcal/mole (two-tailed t-test, not statistically significant at the 5% level).

LOGO representation of the secondary structure demonstrated that nucleotide substitution occurred at one site in the upper stem region (the mature miRNA-miRNA* secondary structure) of pre-miR482, but occurred at four sites in the lower stem region of *Populus* (the RNA-duplex structure adjacent to the mature miRNA-miRNA* stem) (Figure 3A). For pre-miR1448, the nucleotide substitution also did not occur in the upper stem region, but occurred at four sites in the lower stem (Figure 3B).

Thermodynamic profiling of the secondary structure showed that the upper stem of both pre-miR482 and pre-miR1448 had a lower free energy than the lower stem of both pre-miR482 and pre-miR1448. Influenced by nucleotide substitution that occurred at the neighbor nucleotide sites, the thermodynamic profiling of the specific sites (positions 42 and 43 in miR482.2 and positions 34 and 36 in miR1448; see Figure 3) in the lower stem region of the pre-miRNA structure varied among different plants. The variation in thermodynamic profiling in the lower stem of pre-miRNAs implies that it has a function in fine-tuning the processing efficiency of pre-miRNAs [14].

### Substitution ratio of the *MIR482-MIR1448* polycistrons and *rDNA-ITS* regions

To determine the difference in the nucleotide substitution ratio (Kimura 2-parameter) of different regions of these two miRNA genes, the functional and nonfunctional regions were identified in both the *MIR482-MIR1448* polycistrons and *rDNA-ITS* sequences. For the *MIR482-MIR1448* polycistron, the pre-miR482 and pre-miR1448 sequences were the functional region, while 5′ and 3′ flanking sequences of the *MIR482-MIR482* polycistron and internal regions between pre-miR482 and pre-miR1448 sequences were the non-functional regions. For the *rDNA-ITS* sequence, 5.8S rRNA, partial sequences of 18S, and 28S rRNA made up the functional region, whereas the ITS1 and ITS2 regions comprised the non-functional region.

Except for the internal regions of the *MIR482-MIR1448* polycistron, the substitution ratios of the functional regions of the *MIRNAs* were significantly lower than those of the non-functional regions (P value <0.0001, two-tailed t-test) (Table 2). This suggests that the evolutionary rate varied in different regions of the *MIRNAs* and that the functional regions of both poplar *rDNA* and *MIRNAs* were under stronger functional constraints than non-functional regions.

**Table 2:** The substitution ratio of the *MIR482-MIR1448* polycistron and *rDNA-ITS* regions.

Genes	MIR482-MIR1448 polycistron					rDNA-ITS region		
Regions	5′ flanking sequences of pre-miR482	pre-miR482	internal sequences	pre-miR1448	3′ flanking sequences of pre-miR1448	ITS1	Partial sequences of 18S and 28S rDNA, 5.8S rDNA	ITS2
Base pair	92	107	80	85	628	224	221	215
Kimura 2-parameter (Mean±SD)	0.066±0.017	0.014±0.007	0.006±0.003	0.016±0.007	0.060±0.005	0.018±0.005	0.003±0.002	0.019±0.005

The differences of the substitution ratio between functional regions (pre-miR482, pre-miR1448) and nonfunctional regions (5′ and 3′ flanking sequences) were extremely statistically significant (P value <0.0001, two-tailed t-test) and the differences of the substitution ratio between functional regions (pre-miR482, pre-miR1448) and internal regions were very statistically significant (P value <0.01, two-tailed t-test), while the differences of the substitution ratio between pre-miRNAs and ITS (ITS1 and ITS2) were not significant at the 5% level.

The nucleotide substitution ratio also indicated that the evolutionary rates of the two types of RNA were clearly different. The substitution ratio in the functional region of *rDNA* (0.0026) was only 20% of the pre-miRNAs (mean value 0.0155) (Table 2), suggesting that *rDNAs* were under stronger functional constraints than the *MIR482* and *MIR1448* genes. As the integral component in protein synthesis, *rDNA* had a more significant role than the *MIRNA* genes (*MIR482* and *MIR1448*) involved in poplar disease resistance or other stresses at the post-transcriptional level.

**Figure 4:**
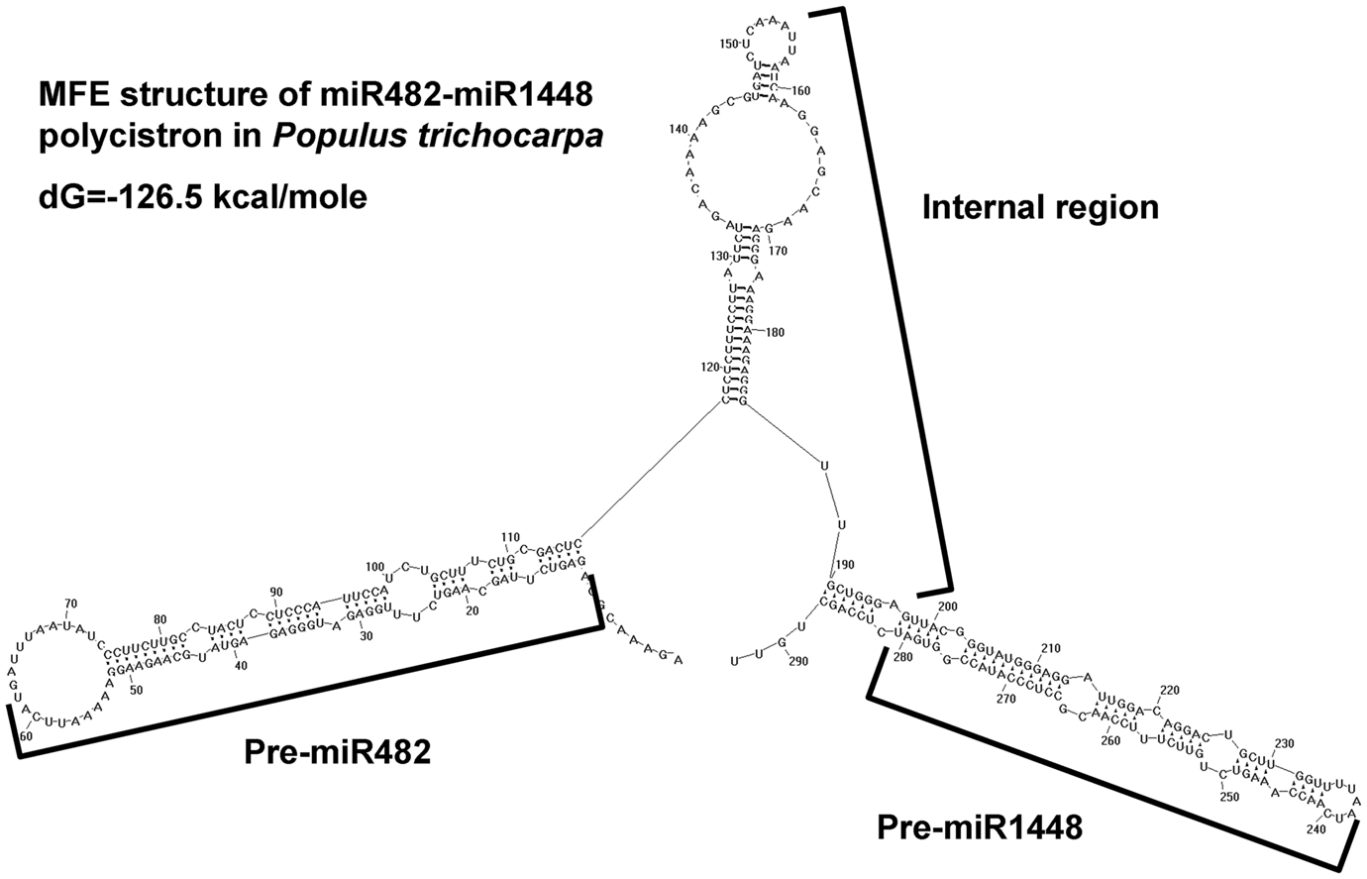
The tertiary structure of miR482-miR1448 polycistron in *Populus trichocarpa*. The MFE structure prediction used RNAstructure v5.3 showed that the pre-miR482 and pre-miR1448 could form a stem-loop structure, meanwhile, the internal region in miR482-miR1448 polycistron also could fold back on itself to form a helix.

There was no difference in the substitution ratio between pre-miR482 and pre-miR1448. However, as mentioned above, the mutation ratio of the internal region was clearly lower than that of the functional regions, and similar to that of *rDNA* regions (Table 2). Recently, Chakraborty et al. (2012) also found that the internal regions in miR-17-92a polycistron are significantly conserved. Moreover, structure analysis shown that the internal regions in miRNAs polycistron, such as that in miR-17-92a polycistron [15], Osa-miR395 and ath-miR774-miR859 polycistron [10], could fold back on itself to form a helix. The MFE structure of poplar *MIR482-MIR1448* polycistrons were predicted in this study. The results also revealed that the internal regions could form a stable stem-loop structure (Figure 4). Therefore, we inferred that these internal regions might have an crucial role in poplar trees. Therefore, we assumed the *MIR482-MIR1448* internal region was not only the union of the two precursors, but with some crucial role in miRNA maturation.

**Figure 5:**
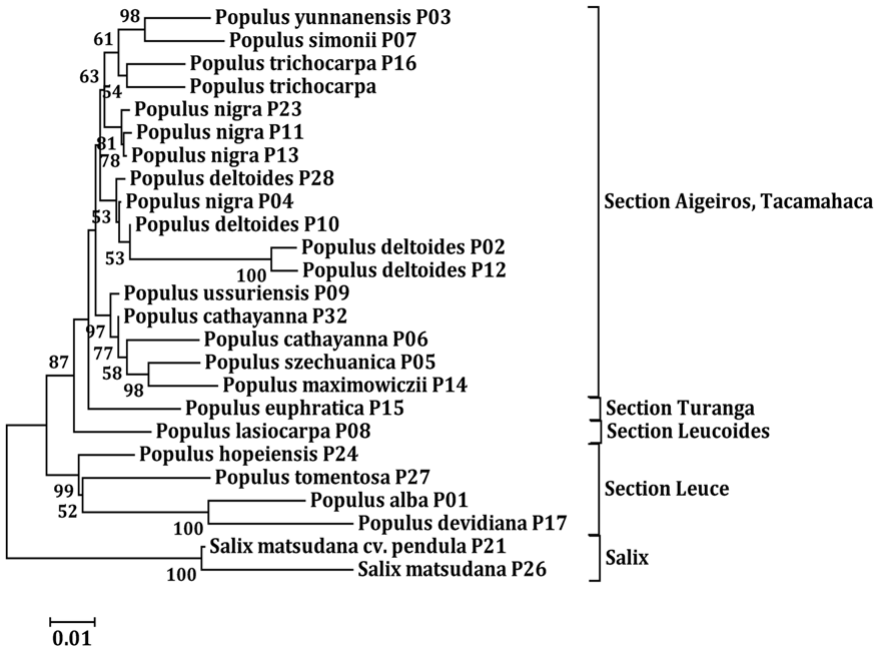
Neighbor-joining phylogenetic trees based on the *MIR482-MIR1448* polycistron and *rDNA-ITS* combined dataset in *Populus*. Numbers in the tree represent the bootstrap value (bootstrap values below 50% are not shown at the nodes). Two *Salix* clones were treated as an outgroup.

In addition, we found that the pre-miRNAs, *rDNA-ITS1*, and *rDNA-ITS2* had similar substitution ratios. Nuclear *rDNA-ITS* sequences have been applied widely to the phylogenetic analysis of plants and fungi [16], [17]. This led to the question of whether the *MIR482-MIR1448* polycistron (the single-copy in the poplar genome) would be beneficial for taxonomic purposes in poplars. We constructed three NJ trees based on the combined data from the *MIR482-MIR1448* polycistron (not including the 5′ upstream sequence before −92 nt from the pre-miR482), *rDNA-ITS* and *MIR482-MIR1448-ITS* data, respectively. The results were similar to the results in the previous study [16]. The three phylogenetic trees could reveal the difference of the species in *Populus* sections *Leuce*, *Turanga*, and *Leucoides*, but could not distinguish the species in sections *Tacamahaca* and *Aigeiros* (the NJ phylogentic tree based on *MIR482-MIR1448-ITS* data see Figure 5).

### Compensatory substitutions in the stem region of pre-miRNAs

To compare the evolutionary rate in the secondary structures of pre-miR482 and pre-miR1448, the model of compensatory substitutions in the stem region of pre-miRNAs was analyzed.

It should be noted that, consistent with strong functional constraints on miRNA secondary structures, the general secondary structures (hairpin and loop structures) are highly conserved in all *MIR482* and *MIR1448* genes in *Populus*, as reflected by the excess of compensatory substitutions over those expected on a purely random basis (Table 3) (single substitutions, significance for *MIR482* at the 0.01% level and significance for *MIR1448* at the 5% level, two-tailed Fisher's exact test). This substitution pattern suggests that compensatory mutations could be the evolutionary mechanism of *MIR482* and *MIR1448*, as in some primate miRNA genes [18] and other functional RNA genes [19], [20].

**Table 3:** Substitutions observed in pre-miR482 and pre-miR1448 in *Populus*, on the basis of the secondary structure of *Populus alba* clone P01.

	Type of substitution	MIR482		MIR1448	
		No. expected	No. observed	No. expected	No. observed
Single substitutions	Base pairing to base pairing (4)	6.3	27	6.9	15
	Base pairing to non-base pairing (28)	43.7	23	40.1	32
Double substitutions	Base pairing to base pairing (11)	0	0	0	0
	Base pairing to non-base pairing (32)	0	0	0	0

The theoretical probability of substitution that converting one pair of complementary bases into another pair of complementary bases has been determined previously (Dixon and Hillis 1993) and listed in parentheses. Single and double substitutions were calculated for pre-miR482 and pre-miR1448 of 33 Populus species (clones) (22 materials in this study and 12 EST from NCBI). Single substitutions, P<0.0001 for MIR482; P<0.05 for MIR1448 (two-tailed Fisher's exact test).

## Discussion

### Origin and evolutionary patterns of the *MIR482* and *MIR1448* genes of *Populus*

In recent years, several models of the origin of *MIRNA* genes have been proposed. It has been thought that inverted duplication of target gene sequences is the main *de novo* generation mechanism of plant *MIRNA* genes [7], [21], [22], [23]. Some novel miRNAs also arise from transposable elements (TEs) or pseudogenes [24], and random sequences [25]. For the miRNA families, they may arise from a process of whole genome duplication (WGD), tandem duplication, or segmental duplication followed by dispersal and diversification, somewhat similar to the processes that drive the evolution of protein gene families [26].

In the present study, based on the phylogenetic analysis of the *MIR482* and *MIR1448* genes, we assumed that *MIR482* was an “old miRNA” gene in seed plants and that *MIR1448* was a “young miRNA” gene in Salicaceae. It has previously been noted that some young *MIRNA* loci originated from one gene family but form miRNAs that target transcripts in another family in *Arabidopsis* [21]. In a similar manner, we suggest that *MIR1448* originated from *MIR482* through tandem replication events in Salicaceae. This leads to a discussion of what mechanisms drove the evolution of replicated *MIR482* into *MIR1448*.

**Figure 6:**
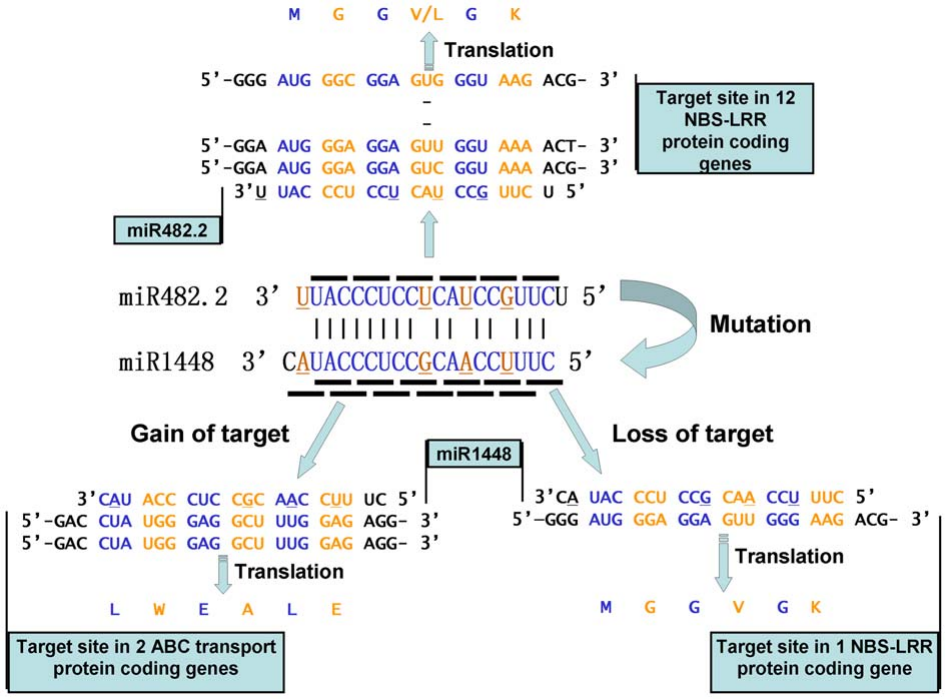
Patterns of gain and loss of target genes in miR1448 miRNAs. For the four nucleotide mutations that occurred in the mature sequence of miR1448 when compared to miR482.2, poplar miR1448 lost the control of most of the target genes (miR482.2 targeted 12 NBS-LRR protein-coding genes while miR1448 only targeted 1 NBS-LRR gene). On the other hand, through a frameshift-targeted mechanism, miR1448 captured two ABC transport protein-coding genes as target genes. The underlined nucleotide site in the miRNAs represents the substitution sites when miR1448 is compared to miR482.2.

Mature sequences of miR482.2 and miR1448 both target the sequences encoding the “MGGV(L)GK” peptide in the NB-ARC domain of some NBS-LRR resistance proteins (Figure 6, Table S1). Although the mature sequences of miR1448 were highly homologous to the mature sequences of miR482.2, four transversional substitutions still occurred in mature miR1448 compared to mature miR482.2. In the miRNA-target complex, we found that the wobble positions in the codons of target genes were very complementary to these four mutant nucleotide sites in the miRNAs, while other nucleotide sites that corresponded to the first and second positions in codons remained stable. Although four tranversional substitutions occurred, there were no transitional substitutions in the mature sequences of the *MIR1448* gene. However, a two-tailed Fisher's exact test illustrated that the ratio of transversion versus transition did not deviate from the theoretical value of natural mutation. Nucleotide mutations play a major role in the gain or loss of miRNA binding sites during evolution [27]. The results of the present study imply that nucleotide mutations in *MIRNA* genes might play a vital role in the formation of the *MIR1448* gene in poplars. Our results also revealed that the first and second positions in codons of target genes of miR1448 should have a purified selection function in the formation of miR1448. Plant miRNA binding sites exhibit almost the exact Watson–Crick complementarity to the entire mature miRNA. In previous research on rice, some miRNAs gained or lost their target genes in association with mutations that occurred in the mature sequences [27]. In the present study, after mutations occurred at specific sites, some target genes were out of the control of miR1448. For example, there were 10 NBS-LRR target genes targeted by miR482.2 while only 1 NBS-LRR gene, POPTR_0019s00620.1, remained as the solemn target gene of miR1448 in poplars (Table S1). The mutated miR1448 also targeted the mRNA region that encoded the “LWEALE” peptide in two ATP-binding cassette transport proteins (ABC transport proteins, such as POPTR_0249s00200.1) and one unknown protein (POPTR_0005s18270.1) through a mechanism that we describe as “frameshift targeted” (Figure 6, Table S1). This confirms that miR1448 captured a new target gene through mutation. With regard to plant ABC transporter proteins associated with polar auxin transport, lipid catabolism, xenobiotic detoxification, disease resistance, and stomatal function [28], this implies that miR1448 might be more significant than miR482.2 in responses to environmental change. Carrington and his colleagues suggested that *MIRNA* genes are undergoing relatively frequent birth and death [21]. In the present study, we suggested that the novel regulatory functions that denoted by the newly captured target genes would benefited *MIR1448* gene escaped from the doom of many young *MIRNA* genes might encountered, and was finally stabilized by integration into poplar regulatory networks.

### Expression patterns of the *MIR482* and *MIR1448* genes

Accumulating evidence suggests that some clustered *MIRNAs* in plants and animals are cotranscribed together as a polycistron [1], [9], [29], [30], [31]. NCBI-EST-Blast also revealed that the whole sequence of pre-miR482 and pre-miR1448 were simultaneously contained in one EST sequence. That is, these two pre-miRNAs could have identical accumulation under the same stress conditions. For example, the expression level of both miR482.2 and miR1448 decrease under cold, heat, and dehydration stresses, and remain stable under salt stress in *P. trichocarpa* [6]. However, the expression of miR1448 is upregulated by at least 1.5 times whereas miR482.2 in poplar remains unchanged under mechanical stress [6]. In a previous study, we also found that miR1448 but not miR482.2 was responsive to fungal pathogen stress in the bark of poplar (unpublished data). However, what caused these differential expression patterns in miR482.2 and miR1448 miRNAs?

Differ from the mRNAs that encoding proteins, another maturation process was needed before the pre-miRNAs mediated gene expression at the post-transcription level. Some analyses in *Arabidopsis* revealed that different argonaute (AGO) proteins select for miRNA with a specific length and 5′ terminal nucleotide [32], [33]. For example, AGO1 binds miRNAs with a 5′U but AGO5 binds miRNAs with a 5′C. Therefore, it was very likely that poplar miR482.2 (with a 5′U) and miR1448 (with a 5′C) might associate with different argonaute proteins, and these two pre-miRNAs might be differentially expressed for their different mature efficiency. Moreover, one study revealed that the tertiary structure of the internal regions in miRNAs polycistron might autoregulate the mature efficiency of the individual miRNAs in difference tissues [15], therefore, the expression level of miR482.2 and miR1448 might also influenced by the tertiary structure of their internal region.

Additionally, it was reported that a single miRNA precursor can produce multiple distinct small RNAs [34]. The pre-miR482 can splice into two miRNAs with a 15nt overlapping region: miR482.1 and miR482.2 [6], [13]. Therefore, only one mature miRNA, either miR482.1 or miR482.2, can be produced in poplar at any given time. This may be the reason for the different expression patterns of the *MIR482* and *MIR1448* under certain stresses. Taking into account the potential difference in the efficiency of mature miRNAs and the differentiation of their target genes, the expression patterns of the clustered *MIRNAs* and even those of the homologous or non-homologous miRNAs in the same ESTs are more complicated than once thought.

In conclusion, we found that the poplar *MIR1448* gene is a tandem duplication product of the *MIR482* gene. Following purified selection from their target genes, nucleotide mutants accumulated at specific sites in the mature sequences of newly formed miR1448. Considering the existence of the Watson–Crick complementarity property between plant miRNA and their binding sites in target genes, it should be noted that here the mutated novel miR1448 partially lost its complementarity to the original targets. This newly formed miRNA further captured another protein-coding gene as a new target. By collaborating with the remaining and newly captured target genes, the *MIR1448* gene became fixed and then integrated into the gene regulatory network of poplars. Although miR1448 was shown to be homologous to miR482, miR1448 acquired some related but not identical functions after a long evolutionary process. Functional analysis of target genes revealed that the major role of miR482 was in resistance to disease or other stresses via NBS-LRR proteins, whereas the biological functions of miR1448 are more diverse.

## Materials and Methods

### Ethics Statement

No specific permits were required for the described field studies. The BBG (Beijing Botanical Garden) is not privately-owned. For the plant materials came from BBG, no specific permissions were required. The other plant materials planted in the greenhouse of CAF (Chinese Academy of Forestry).

### Plant materials and DNA extraction

A total of 26 Salicaceae plants (including 22 *Populus*, 2 *Salix*, and 2 *Idesia*), 3 Euphorbiaceae plants, and 1 Rhizophoraceae plant were used in this study (Table 1). Genomic DNA was extracted from plant leaves using the Takara Universal Genomic DNA extraction kit (Takara, Dalian, China).

### Amplification of the *MIR482-MIR1448* polycistron and *rDNA-ITS* sequences in Salicaceae

To investigate the phylogenetic and evolutionary patterns of the *MIR482* and *MIR1448* genes, the *MIR482-MIR1448* polycistron and their 5′ and 3′ flanking sequences (approximately 400 bp each side, 1100 bp in total) were amplified from each plant. The amplification primers (including five forward primers and seven reverse primers) were designed based on the whole genome sequence of poplar and 12 poplar ESTs that contained both the pre-miR482 and pre-miR1448 sequences (Text S1), using the program Primer 3 version 0.4.0 (http://frodo.wi.mit.edu). The amplified products were directly sequenced on both strands, using these twelve amplification primers with an ABI Prism 3700 DNA Analyzer. In addition, primers ITSL and ITS4 were used to amplify and sequence the entire ITS region of rDNA (including a partial sequence of 18S rDNA, ITS 1, 5.8S rDNA, ITS 2, and a partial sequence of 28S rDNA) in Salicaceae plants. All amplification products were amplified and bi-directionally sequenced independently at least twice to confirm their identity. The primer sequences and their position in the genome sequence of *P. trichocarpa* are listed in Table S2.

### Detection of the MIR482-related polycistron in other plants

To examine whether the miR482-related polycistron was ubiquitously distributed in plants or only was specifically distributed in Salicaceae, the Blast analysis were conducted in the EST and WGS (whole-genome shotgun contigs) database of NCBI using every pre-miR482 sequences of the other plants. Though miR482 was not reported in *Arabidopsis thaliana*, one study showed that miR472 is related to miR482 [8], therefore ath-pre-miR472 was also used to detect miRNAs polycistron structure. And then, companied with the tandem repeated sequence of the queried pre-miR482, the derived ESTs and/or partial WGS nucleotide sequences were alignmented in MUSCLE [35]. Finally, the EST or WGS sequence contained *miR482*-related polycistron was determined according to two criterions: 1), at least one pre-miR482 and one homologous region of this pre-miRNA (the p-distance of these two homologous region was not more 0.40 which determined according to the distance of ptc-pre-miR482 and ptc-pre-miR1448) clustered in one EST sequences, or pre-miR482 and its homologous region was found in a region that not more than 2000 nt in WGS sequence; 2), the homologous region of this pre-miRNA satisfied with the other criterions that used for plant miRNAs predication [36].

### Phylogenetic analysis of plant MIR482 genes

To determine the phylogeny of the *MIR482* gene, we derived all precursor and mature sequences of plant miR482 (Text S2) from miRBase release 18 (http://www.mirbase.org, November, 2011). The pre-miR482 sequences were subjected to multiple sequence alignment using MUSCLE. Neighbor-joining (NJ) phylogenies based on the p-distance and Kimura 2-parameter distance were generated by MEGA version 5.0 [37]. Bootstrap confidence values were obtained applying 1000 replications.

### Secondary structure prediction and thermodynamic profiles of pre-miR482, pre-miR1448 in Salicaceae

The MFE structure of each miRNAs was predicted using the program RNAstructure version 5.3 [38]. The LOGO representation of these structures was obtained with the WebLogo software [39]. The thermodynamic stability profiles of the predicted secondary structures were calculated for pre-miR482 or pre-miR1448 according to the nearest-neighbor method (NNM) [40], and summarized in a single profile by averaging the free energy values at each position.

### Evolution pattern analysis

#### Calculation of the nucleotide substitution ratios of the *MIR482-MIR1448* polycistron and *rDNA-ITS* region

The nucleotide substitution ratio of pre-miRNAs (Kp) and their flanking genomic sequences (Kf) in 22 poplars was calculated using MEGA. The pre-miRNAs were aligned (see Figure S1) using MUSCLE, and the nucleotide substitution parameter (Kimura 2-parameter) was used in this study. Furthermore, the substitution ratios of *rDNA-ITS* sequences were compared to those of *MIR482-MIR1448* clusters in poplars.

#### Calculation of compensatory substitutions in the stem region of pre-miR482 and pre-miR1448

The compensatory substitution ratios in the stem region of pre-miR482 and pre-miR1448 in *Populus* was calculated according to the methods of Zhang et al. [18]. There are two classes of changes that can occur in the stem region of miRNAs: substitutions that change one pair of complementary bases to another pair of complementary bases (e.g., C–G to U–G), and substitutions that change one pair of complementary bases to a pair of non-complementary bases, or vice versa (e.g., C–G to C–C). For a particular site in the stem region, a single substitution refers to a change in either of the two corresponding bases, and a double substitution refers to changes of both bases. The probability of either a single substitution or a double substitution converting one pair of complementary bases into another pair of complementary bases has been determined previously [41]. A total of 22 poplars (Table 1), with 12 poplar EST sequences (Text S1), were analyzed in this study. Pre-miR482 and pre-miR1448 were used to calculate double substitutions (multiple counts were removed) and to test whether compensatory substitutions were overrepresented.

## Supporting Information

Figure S1 **The alignment of miR482-miR1448 polycistron in Salicaceae.**
(TIF)

Table S1 **The target genes of miR482 and miR1448 in** ***Populus trichocarpa*** **.**
(DOC)

Table S2 **The primers used for the amplification and sequencing of miR482-miR1448 polycistrons and rDNA-ITs region in this study.**
(DOC)

Text S1 **12 poplar EST sequences contain miR482-miR1448 polycistron.**
(TXT)

Text S2 **The pre-miR482 and miR482 sequences in miRBase release 18.**
(TXT)
