# Supplementary figures and images for: Phylogenetic Analysis and Molecular Evolution Patterns in the MIR482-MIR1448 Polycistron of Populus L
Source: PLoS One. 2012 Oct 18;7(10):e47811. doi: 10.1371/journal.pone.0047811 (PMC3475693; doi:10.1371/journal.pone.0047811)

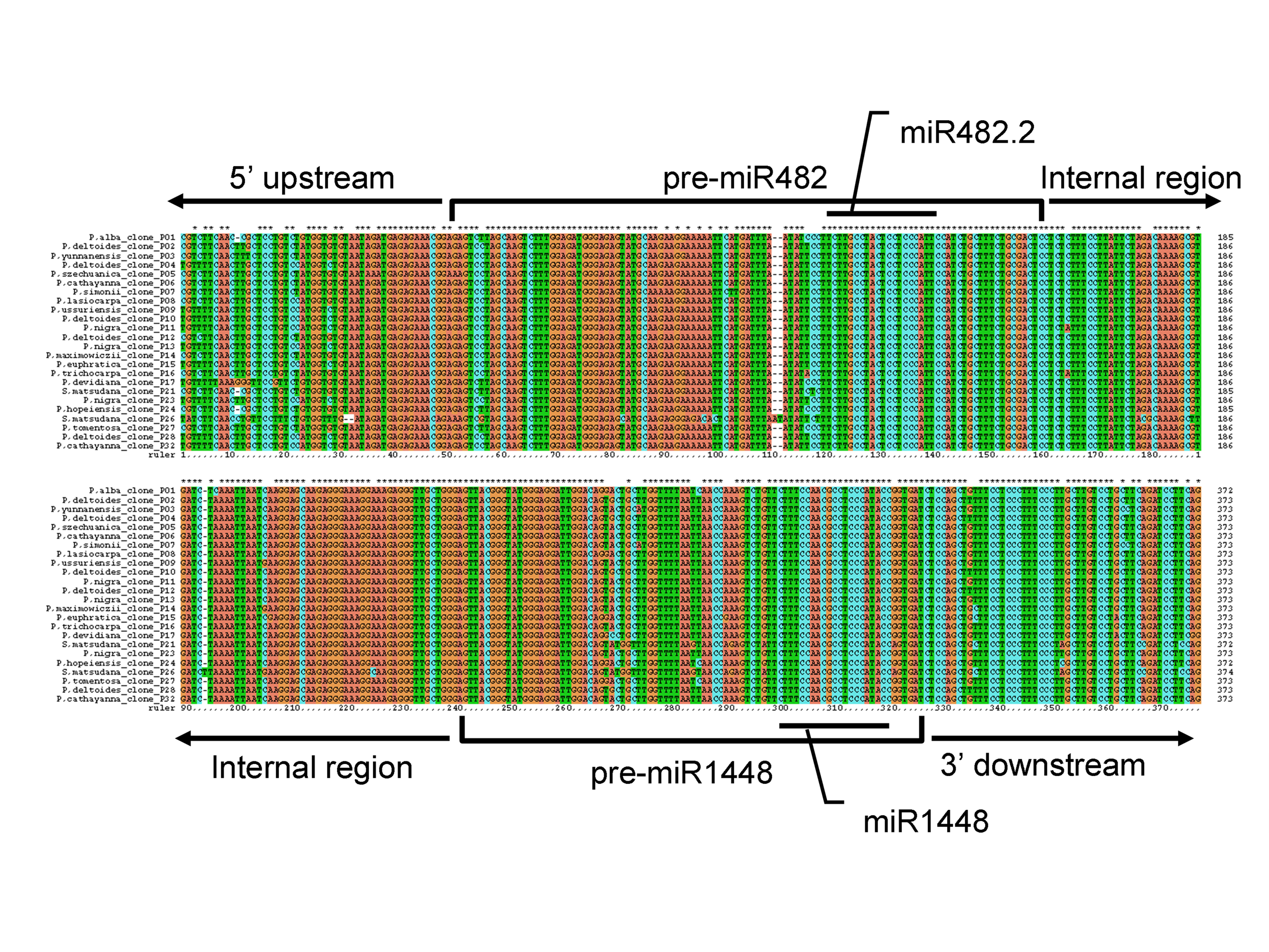

Supplement: Figure S1 — The alignment of miR482-miR1448 polycistron in Salicaceae. (TIF) [file pone.0047811.s001.tif]
