# Supplementary material for: Phylogenetic Analysis and Molecular Evolution Patterns in the MIR482-MIR1448 Polycistron of Populus L
Source: PLoS One. 2012 Oct 18;7(10):e47811. doi: 10.1371/journal.pone.0047811 (PMC3475693; doi:10.1371/journal.pone.0047811)
Supplement: Table S1 — The target genes of miR482 and miR1448 in Populus trichocarpa . (DOC) [file pone.0047811.s002.doc]

Table S1 The target genes of miR482 and miR1448 in *P. trichocarpa* (Lu et al . 2008)

| miRNA | Function | Gene model in JGI Poptr1.1 | Gene model in JGI Poptr2.0 | Target patterns^**^ |
| --- | --- | --- | --- | --- |
| miR1448 | Disease resistance protein | eugene3.01310091 | - | MGGV(L)GK |
|  |  | eugene3.00190077 | POPTR_0019s00620.1^*^ | MGGV(L)GK |
|  | Glutathione S-conjugate ABC transporter (MRP2) | gw1.1700.5.1 | POPTR_0249s00200.1 | LWEALE |
|  | ATP-binding cassette transport protein | gw1.IV.2236.1 | POPTR_0249s00200.1 | LWEALE |
|  | Unknown | fgenesh4_pg.C_LG_V000530 | POPTR_0005s18270.1 | LWEALE |
| miR482 | Disease resistance protein | eugene3.00102261 | - | MGGV(L)GK |
|  |  | eugene3.00190017 | POPTR_0019s01010.1 | MGGV(L)GK |
|  |  | gw1.8759.5.1 | POPTR_0017s01420.1 | MGGV(L)GK |
|  |  | gw1.VI.1923.1 | POPTR_0006s14910.1 | MGGV(L)GK |
|  |  | eugene3.00190077 | POPTR_0019s00620.1^*^ | MGGV(L)GK |
|  |  | eugene3.00180517 | POPTR_0018s02660.1 | MGGV(L)GK |
|  |  | eugene3.00440220 | POPTR_0017s01420.1 | MGGV(L)GK |
|  |  | grail3.0085005401 | POPTR_0019s03720.1 | MGGV(L)GK |
|  |  | eugene3.01170064 | POPTR_0019s01670.1 | MGGV(L)GK |
|  |  | fgenesh4_pg.C_LG_XIX000056 | - | MGGV(L)GK |
|  |  | grail3.0140004801 | POPTR_0011s16850.1 | MGGV(L)GK |
|  |  | fgenesh4_pg.C_scaffold_7992000001 | POPTR_0019s00540.1 | MGGV(L)GK |
|  | Unknown | fgenesh4_pg.C_LG_VI001152 | POPTR_0006s20610.1 | MGGV(L)GK |

Note:

*. The target gene that overlap miR482 and miR1448;

**. The encoded peptide in miRNAs target sites in the predicted target genes of miR482 and miR1448 in *P. trichocarpa*.
