# Supplementary material for: Phylogenetic Analysis and Molecular Evolution Patterns in the MIR482-MIR1448 Polycistron of Populus L
Source: PLoS One. 2012 Oct 18;7(10):e47811. doi: 10.1371/journal.pone.0047811 (PMC3475693; doi:10.1371/journal.pone.0047811)
Supplement: Table S2 — The primers used for the amplification and sequencing of miR482-miR1448 polycistrons and rDNA-ITs region in this study. (DOC) [file pone.0047811.s003.doc]

Table S2. The primers used for the amplification and sequencing of *MIR482-MIR1448* polycistron and *rDNA-ITs* region in this study.

| Gene | Primers | Primer sequence | Position in *P. trichocarpa* clone Nisqually-1 (Tuskan et al. 2006) | |
| --- | --- | --- | --- | --- |
|  |  |  | start | end |
| miR482-miR1448 polycistron | MF520 | 5’-CAGGTCCACTCTCCACTCTC-3’ | -520 | -500 |
|  | MF1 | 5’-CAAGTCTTTGGAGATGGGAG-3’ | +10 | +29 |
|  | MF2 | 5’-AGATGGGAGAGTATGCAAGAAG-3’ | +21 | +42 |
|  | MFn1 | 5’-GCTCCTGTCTRTGGTGTGTA-3’ | -38 | -19 |
|  | MFn2 | 5’-GAAACGGAGAGTCCTAGCA -3’ | -8 | +11 |
|  | MR606 | 5’-AAACAAAGCTGACCACACCC-3’ | +878 | +858 |
|  | MR428 | 5’-CAGCATCCCCGGAATCGA-3’ | +700 | +683 |
|  | MR6 | 5’-ACGATACTCCATAACCAACAACTC-3’ | +538 | +515 |
|  | MR408 | 5’-AGGCGCGAACAGATATGTTG-3’ | +680 | +661 |
|  | MF1R | 5’-CTCCCATCTCCAAAGACTTG-3’ | +29 | +10 |
|  | MF2R | 5’-CTTCTTGCATACTCTCCCATCT-3’ | +42 | +21 |
|  | MR9 | 5’-TCCATAACCAACAACTCGCT-3’ | +531 | +512 |
| rDNA-ITs region | I TSL | 5’-CGTAACAAGGTTTCCGTAGG-3’ |  |  |
|  | ITS4 | 5’-TCCTCCGCTTATTGATATGC-3’ |  |  |

Note: “-520” indicate that at the 520 site before the first nucleotide of pre-miR482 sequence, and “+10” indicate that at the 10 site from the nucleotide of pre-miR482 sequence.
